# Crossed cerebellar diaschisis on CT perfusion in large vessel occlusion stroke: early predictors and clinical relevance in the hyperacute phase

**DOI:** 10.1007/s00415-026-13750-z

**Published:** 2026-03-24

**Authors:** Marcello Naccarato, Edoardo Ricci, Giovanni Furlanis, Katerina Iscra, Francesca Dal Molin, Michele Malesani, Gabriele Prandin, Emanuele Vincis, Magda Quagliotto, Gianpiero Farina, Paola Caruso, Maja Ukmar, Miloš Ajčević, Paolo Manganotti

**Affiliations:** 1 Department of Medicine, Surgery and Health Sciences, Neurology Unit, University Hospital and Health Services of Trieste, University of Trieste, Strada di fiume, 447, 34149 Trieste, Friuli-Venezia Giulia Italy; 2 Department of Engineering and Architecture, University of Trieste, Trieste, Friuli-Venezia Giulia Italy; 3 Radiology Unit, Department of Medicine, Surgery and Health Sciences, University Hospital and Health Services of Trieste - ASUGI, University of Trieste, Trieste, Friuli-Venezia Giulia Italy

**Keywords:** Crossed cerebellar diaschisis, CT perfusion, Functional outcome, Acute ischemic stroke, Functional neuroimaging

## Abstract

**Background and purpose:**

Crossed cerebellar diaschisis (CCD) is a pathophysiological phenomenon in ischemic stroke (IS) that remains poorly investigated, particularly with regard to its clinical impact and the factors associated with its occurrence during the hyper-acute phase. The aim of this study was to evaluate the prevalence of CCD detected by Computed Tomography Perfusion (CTP) in patients with large vessel occlusion (LVO) ischemic stroke and to identify potential predictors of CCD occurrence and its impact on clinical outcomes.

**Materials:**

Clinical and radiological data were collected and analyzed from 256 consecutive patients with anterior circulation LVO ischemic stroke who underwent CTP. The presence of CCD was assessed through qualitative analysis of CTP perfusion maps. Univariate and multivariate logistic regression analyses were performed to identify factors associated with the presence of CCD, as well as to determine predictors of clinical outcome.

**Results:**

Perfusion alterations consistent with CCD were identified in 216 patients (84.4%). In multivariable analysis, female sex (p = 0.026) and greater supratentorial hypoperfused volume assessed on mean transit time (MTT) maps (p = 0.005) were independently associated with the presence of CCD. Although CCD was associated with a higher prevalence of unfavorable functional outcome at 3 months (mRS 3–6) in univariate analysis, it was not an independent predictor of outcome in multivariable analysis.

**Conclusions:**

CCD was detected on CTP in a high proportion of patients with LVO stroke. Female sex and larger supratentorial hypoperfused volumes were independent predictors of CCD. Although correlated to functional outcome, CCD was not an independent predictor.

**Supplementary Information:**

The online version contains supplementary material available at 10.1007/s00415-026-13750-z.

## Introduction

Diaschisis refers to a functional depression in a structurally intact brain region that is anatomically connected to a distant site of injury, such as a stroke [1, 2]. Crossed cerebellar diaschisis (CCD) is characterized by reduced perfusion and metabolic activity in the cerebellar hemisphere contralateral to a supratentorial lesion [1, 3]. The most widely accepted mechanism involves deafferentation of the cortico-ponto-cerebellar pathway, leading to secondary functional impairment within the cerebellum [4]. First described more than a century ago, CCD has since been documented with several neuroimaging modalities, including positron emission tomography (PET), single-photon emission computed tomography (SPECT) and functional magnetic resonance imaging (fMRI), with reported prevalence ranging from 13.4% to 46.2% [5–8].

The use of CT perfusion (CTP) in the management of ischaemic stroke is becoming increasingly common, as it has enabled the extension of the reperfusion treatment window, particularly in patients with large vessel occlusion (LVO) [9, 10] and in wake-up stroke [11, 12]. Furthermore, CTP has demonstrated moderate accuracy in identifying perfusion abnormalities within the posterior circulation and infratentorial stroke [13, 14]. Recent CTP studies reporting a CCD prevalence from 31% to 70.9% of patients with anterior circulation stroke in the acute phase [15–18]. Although the occurrence of CCD has been widely documented using CTP in the acute phase of anterior circulation stroke, the clinical variables associated with its presence remain incompletely characterized, and its association with clinical outcome has not yet been clearly established. Furthermore, research addressing exclusively patients with anterior circulation LVO evaluated by CT perfusion, and excluding medium or distal vessel occlusions, remains limited.

The present study aimed to assess the occurrence of CCD detected by CT perfusion in the acute phase of a large cohort of anterior circulation LVO stroke, to identify clinical and radiological predictors associated with its presence and to explore its potential role as predictor of functional outcome, measured by the modified Rankin Scale (mRS) at 3 months.

## Materials and methods

### Study population

A retrospective analysis was performed on clinical and neuroimaging data from patients with anterior circulation LVO acute ischemic stroke admitted to the Stroke Unit of University Hospital of Trieste between April 2016 and July 2023 who underwent CTP evaluation within 24 h since symptoms onset. On admission, a multimodal CT protocol was performed, comprising non-enhanced CT (NECT), CT angiography (CTA) of the extra- and intracranial vessels and CTP. All patients underwent a control NECT from 24 to 72 h from stroke onset. No restrictions were applied regarding age or gender for inclusion. All types of treatment, including thrombolysis, thrombectomy, or medical treatment, were considered in the study according to the current guidelines [19]. Patients were excluded if they had: (1) medium vessel occlusion (MEVO); (2) distal vessel occlusion; (3) lacunar stroke (small vessel occlusion - SVO); (4) posterior circulation stroke; (5) hemorrhagic stroke; and (6) stroke mimics.

All patients underwent a comprehensive diagnostic workup, including assessment of vascular risk factors, neurological examination, laboratory tests, and instrumental investigations (electrocardiogram, carotid ultrasonography, transthoracic or transesophageal echocardiography, Holter ECG monitoring, electroencephalography, and magnetic resonance imaging) to determine the stroke mechanism.

The research was conducted in line with the principles of the Declaration of Helsinki. The study was approved by the Local Ethics University Committee (University of Trieste Ehitcs Committee).

### Clinical data

Among the clinical data collected, we recorded demographic information (age, sex) and vascular risk factors, including atrial fibrillation (AF), diabetes mellitus (DM), arterial hypertension (AH), smoking status, dyslipidemia, chronic heart failure, history of coronary artery disease, peripheral vascular disease, chronic kidney disease (defined as an estimated glomerular filtration rate [eGFR] < 45 mL/min, calculated using the Cockcroft–Gault equation [20]) and previous ischemic stroke.

Stroke etiology was classified according to the TOAST (Trial of Org 10172 in Acute Stroke Treatment) criteria [21]. The National Institutes of Health Stroke Scale (NIHSS) score was recorded at both admission and discharge [22]. Functional status was assessed using the modified Rankin Scale (mRS) prior to stroke, at discharge, and at 3 month follow-up [23]. The mRS at 3 months was performed either during a scheduled in-person follow-up visit or, when patients were unable to attend the hospital, via a structured telephone interview. The One-year mortality rate was calculated as the proportion of patients who died during their follow-up. Recanalization after endovascular thrombectomy (EVT) was assessed according to the mTICI scale [24]. Timing was measured from onset to hospital admission (onset-to-door), CT perfusion (onset-to-CTP), and reperfusion at the end of thrombectomy (onset-to-end of thrombectomy).

### CT acquisition, protocol and post-processing

Imaging was performed with one of the latest generation CT scanners (Brilliance iCT 256 slices; Philips Medical Systems, Best, Netherlands). CTP acquisition protocol involved intravenous injection of 75 mL of contrast medium, followed by a 40 mL of saline bolus, both administered at an injection rate of 4 mL/s. The exposure parameters used were 80 kVp and 150–200 mAs and three-dimensional axial acquisitions on a whole brain volume with a reconstruction of the slices set to 5 mm were performed using a series of repeated movements of the scanner table. The acquisitions were carried out every 4 s, resulting in a total scanning time of 60 s. CTP data were processed using the Extended Brilliance Workstation v4.5 (Philips Medical Systems).

Perfusion maps, including mean transit time (MTT), cerebral blood volume (CBV) and cerebral blood flow (CBF), were generated using deconvolution-based methods. Time-attenuation curves were modeled via least squares curve fitting. MTT was computed through a closed-form deconvolution of the voxel-specific time/concentration curve using the arterial input function. CBV was determined as the area under the time/concentration curve for each voxel, and CBF was derived as the ratio of CBV to MTT. Radiological studies were analyzed and evaluated in acute cases by a certified neuroradiologist (MU). In cases of uncertain diagnostic interpretation, the results were examined and discussed jointly by the neuroradiologist and two expert neurologists from the Stroke Unit (GF, PC) to reach a consensus. Non-contrast CT (NECT) at admission was assessed using the Alberta Stroke Program Early CT Score (ASPECTS) [25], while collateral circulation on CTA was graded according to Tan’s classification [26]. The total hypoperfused volume was derived from mean transit time (MTT) maps, while the ischaemic core volume was calculated from cerebral blood volume (CBV) maps, and both expressed in milliliters (mL). Specific threshold values were set up in the software to identify the penumbra areas (MTT > 7 s or 145% of the contralateral healthy area and CBV > 2.0 mL/100 g). Final infarct volume was assessed on follow-up non-enhanced CT (NECT) using manual estimation with the ABC/2 method [27, 28]. CCD was identified by qualitative visual assessment, defined as contralateral cerebellar hypoperfusion characterized by alteration in at least one map, persisting across at least three consecutive axial slices and differing clearly from the symmetrical contralateral region. This hypoperfusion pattern was defined by the presence of alterations in at least one perfusion map, characterized by increased MTT or TTP and/or reduced CBV or CBF in the affected cerebellar region. The number of cases with perfusion abnormalities on the CTP summary map was also assessed. The degree of diaschisis was semi-quantitatively classified as mild–moderate or moderate–severe, based on visual detectability, the extent of color scale variation, and the number of consecutive slices showing the abnormality. More pronounced chromatic changes, wider spatial distribution, and greater slice-to-slice consistency were indicative of higher severity.

### Statistical analysis

We performed all statistical analyses using MATLAB (MathWorks Inc., Natick, MA). Descriptive statistics were presented as medians and interquartile ranges (IQR) for continuous variables, and as counts and percentages (n, %) for categorical variables. Differences between stroke patients with and without CCD were evaluated using the Mann–Whitney U test for continuous variables and the chi-square test for categorical variables. A value of p < 0.05 was considered as significant. Univariate and multivariable logistic regression analyses were performed on a predefined subset of variables [sex, age (per 10 year increase), NIHSS at baseline, AF, DM, AH, CHF, dyslipidemia, hypoperfused volume (MTT, per 10 mL increase), core volume (CBV, per 10 mL increase), and lesion side] to identify predictors of CCD occurrence. Variables showing a p-value < 0.05 in univariate analyses were subsequently entered into the multivariable model.

Spearman’s rank correlation analysis was then used to assess the association between baseline NIHSS score and CCD severity (absent, mild–moderate, and moderate–severe).

In addition, univariate and multivariable logistic regression analyses were performed to identify predictors of unfavorable functional outcome, defined as a mRS score of 3–6 at 3 months. The following variables were included: sex, age, NIHSS at baseline, AF, DM, AH, dyslipidemia, CHF, hypoperfused volume (MTT), core volume (CBV), and the presence of CCD.

Subgroup analyses were conducted in patients who did not undergo mechanical thrombectomy and in patients without neurological improvement, defined as a change in NIHSS ≤ 5 during hospitalization, using the same set of covariates. Analyses were subsequently repeated in patients with CCD only, replacing the presence of CCD with its severity.

Odds ratios (OR) with corresponding 95% confidence intervals (CI) were calculated for all logistic regression models.

## Results

**Fig. 1:**
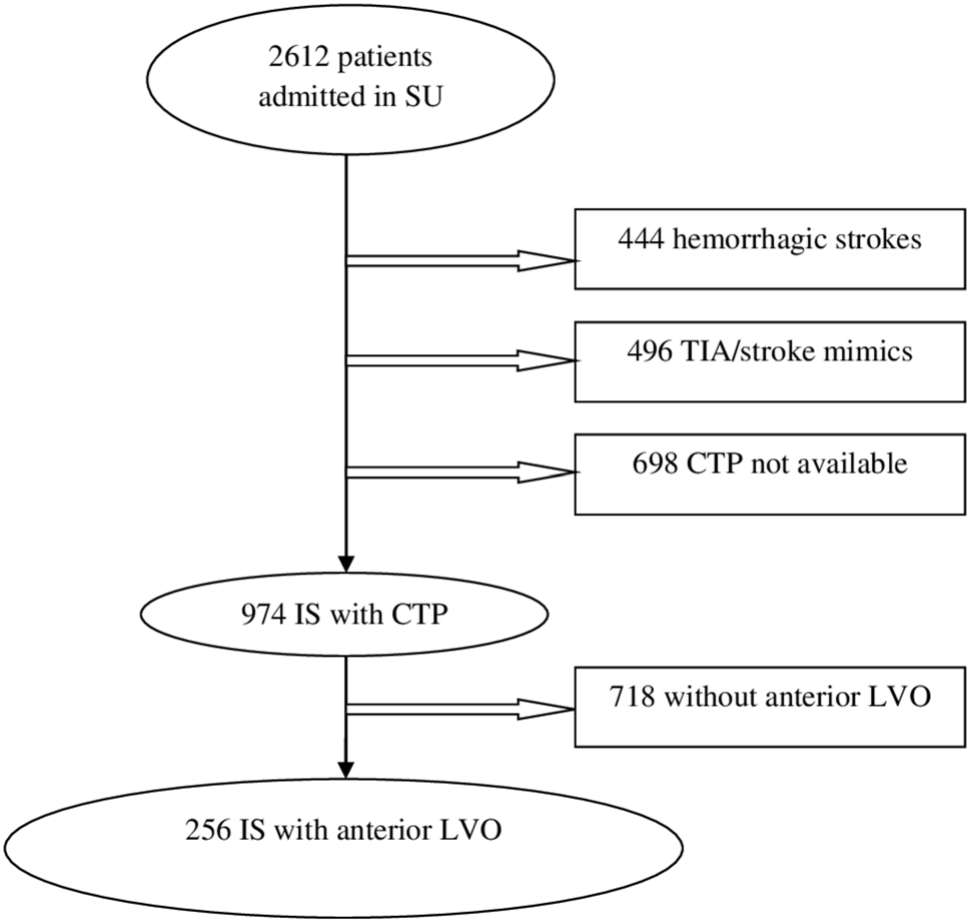
Study flow diagram

From April 2016 to July 2023, 2612 patients were admitted to the Stroke Unit. Of these, 256 patients with anterior circulation large vessel occlusion underwent acute assessment with CT perfusion and were included in the study. The study flowchart is shown in Fig. 1. Baseline clinical, demographic, and radiological characteristics as well as comparison between CCD+ and CCD− groups are summarized in Table 1, while main CCD perfusion characteristics are reported in Table 2.

**Table 1:** Comparison of demographics and clinical characteristics of patients with crossed cerebellar diaschisis (CCD) vs patients without CCD

	n = 256	CCD present n = 216	CCD absent n = 40	*p-values*
**Demography**				
Age (years) [median (IQR)]	78 (71–84)	79 (72–84)	75.5 (65.8–80)	**0.017**
Females [n (%)]	152 (59.4)	133 (61.6)	19 (47.5)	**0.036**
**Comorbidities** [n (%)]				
Atrial fibrillation	117 (45.5)	103 (47.7)	14 (35)	0.139
Diabetes mellitus	47 (18.4)	37 (17.1)	10 (25)	0.238
Arterial hypertension	194 (75.8)	163 (75.5)	31 (77.5)	0.782
Smoking status	44 (17.2)	34 (15.7)	10 (25)	0.154
Dyslipidaemia	134 (52.3)	109 (50.5)	25 (62.5)	0.161
Chronic heart failure	38 (14.8)	32 (14.8)	6 (15)	0.976
Coronaropathy	44 (17.2)	34 (15.7)	10 (25)	0.154
Peripheral vascular disease	41 (16)	37 (17.1)	4 (10)	0.259
Chronic kidney disease	31 (12.1)	28 (13)	3 (7.5)	0.331
Cognitive impairment	22 (8.6)	20 (9.3)	2 (5)	0.377
Previous ischemic stroke	22 (8.6)	18 (8.3)	4 (10)	0.730
**TOAST classification** [n (%)]				
Cardioembolic (CE)	115 (44.9)	100 (46.3)	15 (42.5)	0.477
Large artery atherosclerosis (LAA)	40 (15.6)	32 (14.8)	8 (20.0)	0.608
Undetermined causes (SUC)	80 (31.2)	70 (32.4)	10 (25.0)	0.735
Other determined causes (ODC)	21 (8.2)	14 (6.5)	7 (17.5)	0.653
Small vessel occlusion (SVO)	0 (0.0)	0 (0.0)	0 (0.0)	//
**Stroke severity and clinical outcomes**				
NIHSS at baseline	15 (9–21)	15 (9–21)	11 (6–20)	0.103
NIHSS at discharge	4 (1–14)	4 (1–15)	1 (0–7)	0.086
NIHSS at discharge ≤ 5	127 (49.6)	102 (47.2)	25 (62.5)	0.076
Pre-stroke mRS	0 (0–1)	0 (0–1)	0 (0–1)	0.596
mRS at 3 months	3 (1–5)	4 (1–5)	2 (1–5)	**0.026**
mRS at 3 months 0–1	74 (28.9)	58 (26.9)	16 (40)	0.092
mRS at 3 months 0–2	101 (39.5)	79 (36.6)	22 (55)	**0.029**
One-year mortality	85 (33.2)	77 (35.7)	8 (20)	0.054
Length of hospitalization	9 (5–17)	10 (5–17)	7 (5–12)	0.297
**Reperfusion treatments** [n (%)]				
Thrombectomy	35 (13.7)	30 (13.9)	5 (12.5)	0.814
Thrombolysis	44 (17.2)	34 (15.7)	10 (25)	0.154
Thrombolysis and thrombectomy Recanalization score	147 (57.4)	127 (58.8)	20 (50)	0.301
TICI < 2b	45 (24.7)	40 (25.5)	5 (20)	0.358
TICI 2b-3	137 (75.3)	117 (74.5)	20 (80)	0.358
Timing				
Onset to door (hours)	1.28 (0.57–2.50)	1.27 (0.56–2.46)	1.35 (0.59–2.57)	0.781
Onset to first CTP (hours)	1.57 (1.20–3.10)	1.55 (1.21–2.55)	2.15 (1.21–3.54)	0.362
Onset to end of thrombectomy (hours)	2.35 (2.05–3.12)	2.37 (2.05–3.12)	2.25 (2.10–2.58)	0.380
**Neuroimaging**				
NECT at admission				
ASPECTS [median (IQR)] CTA at admission	9 (8–10)	9 (8–10)	10 (8–10)	0.740
Collateral score [median (IQR)]	2 (1–2)	2 (1–2)	2 (1–3)	0.361
CTP at admission				
Hypoperfused volume (mL)	112.3 (64.6–170.6)	121.4 (66.8–181.7)	77.8 (42.3–144.9)	**0.030**
Core volume (mL)	15 (5.8–42.1)	17.2 (6.2–45.3)	10.7 (3.1–26.9)	0.133
NECT at follow-up				
Final infarct volume (mL)	14.7 (2.4–75.5)	16.8 (2.4–78.9)	11.22 (1.5–26.3)	0.200
Lobar involvement	208 (81.3)	176 (81.5)	32 (80)	0.825
Side				**0.004**
Right	110 (43)	101 (46.8)	9 (22.5)	
Left	146 (57)	115 (53.2)	31 (77.5)	
Cerebral infarcted area [n (%)]				
Affected cerebral lobes [median (IQR)]	2 (1–4)	2 (1–4)	2 (1–3)	0.081

Data are presented as medians (IQR), and frequencies when appropriated

Bold values for *p* < 0.05

**Table 2:** Characteristics of hypoperfusion in patients with crossed cerebellar diaschisis

	n = 256
**Evidence of CCD**	
Degree of CCD [n (%)]	
Mild to moderate	178 (69.5)
Moderate to severe	38 (14.8)
Perfusion maps showing CCD [n (%)]	
MTT	167 (65.2)
TTP	87 (34.0)
CBV	194 (75.8)
CBF	188 (73.4)
Perfusion map with best CCD visualization	
MTT	42 (16.4)
TTP	25 (9.8)
CBV CBF	93 (36.3) 56 (21.9)

Perfusion alterations consistent with crossed cerebellar diaschisis were identified in 216 out of 256 included patients (84.4%). Diaschisis was mild-to-moderate in 69.5% of cases and moderate-to-severe in 14.8%. CCD was most frequently detected on CBV maps (75.8%), followed by CBF maps (73.4%).

Compared with patients without CCD, those with CCD were older (median 79 vs. 75.5 years, p = 0.017) and more frequently female (61.6% vs. 47.5%, p = 0.036). Baseline NIHSS scores were higher in patients with CCD (median 15 [9–21] vs. 11 [6–20]; p = 0.103), although the difference did not reach statistical significance; a trend toward higher NIHSS scores at discharge was also observed (median 4 [1–15] vs 1 [0–7]). Moreover, CCD-positive patients had larger supratentorial hypoperfused volumes on CT perfusion (121.4 vs. 77.8 mL, p = 0.030). Functional outcomes were poorer in the CCD group, with higher 3-month mRS scores (4 [1–5] vs. 2 [1–5], p = 0.026), lower rates of good outcome (mRS 0–2: 36.6% vs. 55%, p = 0.029).

A comparison of clinical and neuroimaging data between patients with mRS 0–2 at three months and mRS 3–6 is available in Supplementary material.

Finally, Spearman’s rank correlation analysis showed a significant, although weak association, between baseline NIHSS score and CCD severity (absent, mild–moderate, moderate–severe; ρ = 0.15, p = 0.017). The distributions of NIHSS scores at admission across CCD severity categories, depicted as violin plots, are available in Supplementary material.

Regarding the features that are associated with CCD presence, the univariate regression analysis identified female sex, higher age, atrial fibrillation and higher hypoperfused volume (MTT/10) as significant parameters associated with the presence of CCD. When the significant variables were included in the multivariate analysis, it was found that female sex (OR: 1.284, p = 0.026), and higher hypoperfused volume (MTT/10) (OR: 1.017; p = 0.005) are independent predictor of presence of CCD (Table 3).

**Table 3:** Logistic multivariate regression for prediction of occurrence of CCD

Variables	OR	Cl–95%	*p-values*
Female sex	1.284	1.029—1.602	**0.026**
Age (per 10 year increase)	1.065	0.977—1.161	0.146
Atrial fibrillation	1.093	0.948—1.260	0.213
Hypoperfused volume (MTT) (per 10 mL increase)	1.017	1.005—1.029	**0.005**

Multivariate analysis for prediction of occurrence of CCD. Bold values for *p* < 0.05

Analysis of the predictive value of CCD, alongside other clinical and radiological variables, showed that in the overall cohort, univariate analysis identified female sex, age, admission NIHSS score, diabetes mellitus, hypertension, chronic heart failure, hypoperfused volume on MTT, core volume on CBV, and the presence of CCD on CTP as significant. In the multivariable logistic regression analysis older age (OR: 1.008; p = 0.002), higher NIHSS score at admission (OR: 1.031; p < 0.001), DM (OR: 1.178; p = 0.016), and CHF (OR: 1.123, p = 0.032) emerged as independent predictors of unfavorable functional outcome at 3 months (mRS 3–6).

In a separate multivariable analysis focused only to patients with CCD, age (OR: 1.009; p < 0.001) and baseline NIHSS (OR: 1.027; p < 0.001) remained independently associated with poor functional outcome, whereas perfusion-derived volumetric parameters and the degree of diaschisis were not independently predictive**.**

Additional subgroup analyses were performed to account for the potential confounding effects of reperfusion treatment and early neurological improvement. In patients who did not undergo mechanical thrombectomy, age (OR: 1.012; p = 0.016) and baseline NIHSS (OR: 1.032; p < 0.001) were independent predictors of unfavorable outcome, while CCD was not independently associated with 3 month mRS. In contrast, among patients without early neurological improvement during hospitalisation (ΔNIHSS ≤ 5), CCD (OR: 1.164; p = 0.042) remained independently associated with unfavorable functional outcome, together with baseline NIHSS (OR: 1.034; p < 0.001)**.**

The details regarding these analysis are reported in Supplementary material.

## Discussion

The main finding of this study is that CT perfusion can identify perfusion abnormalities consistent with CCD in the hyperacute phase of anterior circulation LVO stroke; this phenomenon was observed on CTP in 84.4% of cases. Among perfusion parameters, CBV and CBF showed the highest detection rates. CCD was more frequently observed in female patients and in those with larger supratentorial hypoperfusion volumes, as assessed by MTT volume.

These findings were obtained in a homogeneous population, as our study exclusively focused on strokes caused by anterior circulation LVO, whereas previous investigations also included medium and distal middle cerebral artery occlusions [17] and, in some cases, posterior cerebral artery occlusions [16]. Moreover, the wide variability in CCD prevalence reported across studies is likely attributable to methodological heterogeneity, including small sample sizes, differences in vascular territories, the inclusion of both acute and chronic stroke populations, and the use of different imaging modalities, such as PET, SPECT, and fMRI, with differing sensitivities for its detection [5–8, 29].

In our cohort, the prevalence of observed CCD on CTP was higher than that reported in a previous CTP study conducted in the acute phase of MCA territory infarction (35.3%) [15], including a control cohort which consisted of patients, without supratentorial cerebrovascular hypoperfusion pattern, with a diagnosis of transient ischaemic attacks, epileptic seizures, inflammatory conditions of the central nervous system, encephalopathy, and intracerebral hemorrhage. In this control group cerebellar perfusion abnormalities were observed in 12.5% [15]. However, our findings are consistent with a PET-based study that included a healthy control group, in which CCD was quantitatively identified in up to 89% of patients with MCA occlusion [29].

In our study focusing on patients with LVO strokes, conditions typically associated with more extensive cerebral injury and greater disruption of the cortico-ponto-cerebellar pathways, we included a population more prone to developing the CCD phenomenon, which likely increased its detectability. Generally, the sensitivity of CT perfusion to hemodynamic alterations, combined with its rapid acquisition time, enables reliable assessment even in the hyperacute phase of ischemic stroke, allowing observation of this phenomenon at early stages. Moreover, the results obtained in this study further provide evidence that CTP is able to detect perfusion abnormalities in the posterior cranial fossa, not only in cases of posterior circulation stroke (PCS) [13, 14], but also in instances of functional disconnection secondary to supratentorial stroke.

**Fig. 2:**
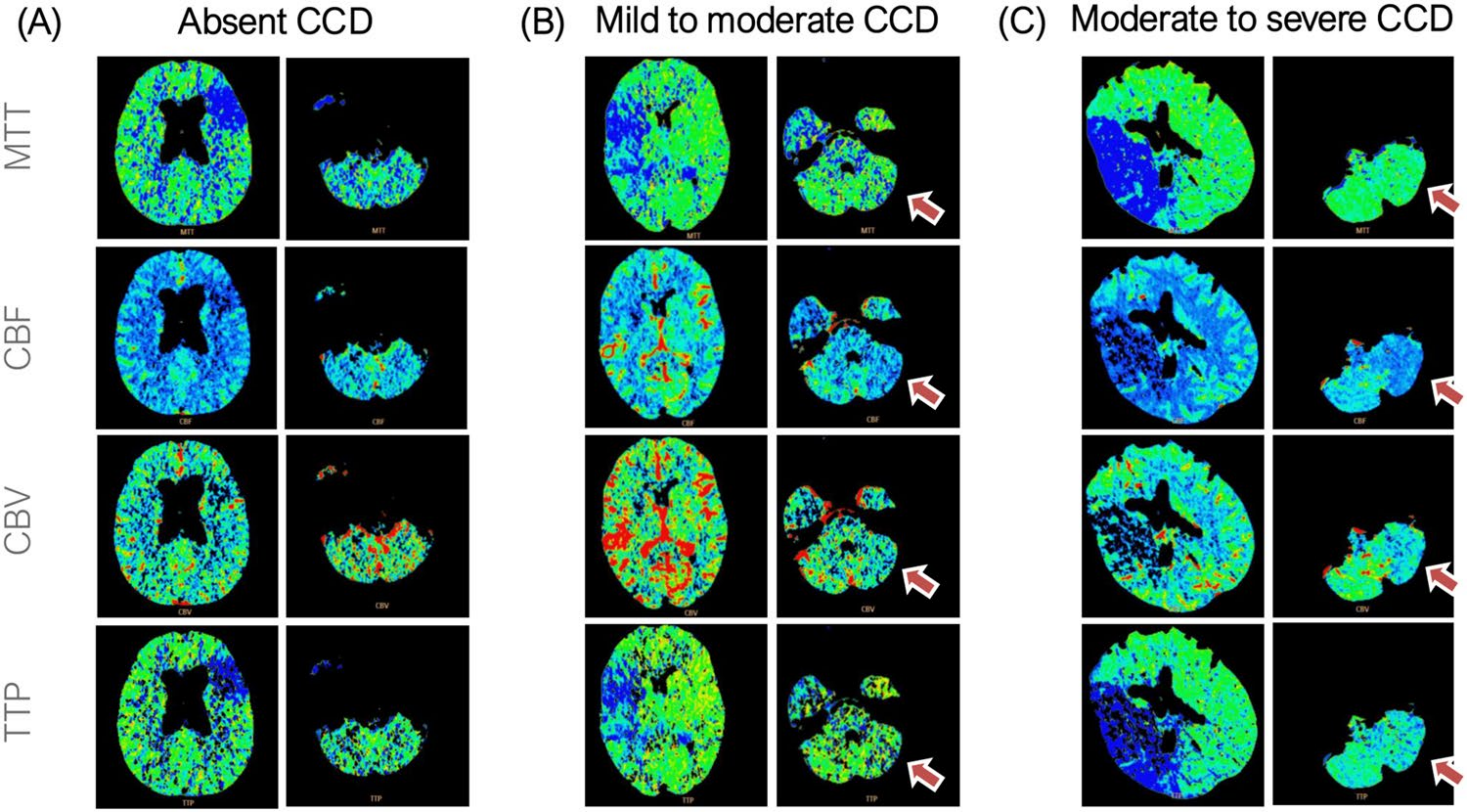
Representative cases of absence of CCD, mild to moderate and moderate to severe CCD. **A** A 77 year-old man presented to the emergency department two hours after symptom onset with right-hand weakness and motor aphasia (baseline NIHSS: 3). Imaging revealed an occlusion of the left M2 segment of MCA, and CT perfusion demonstrated a hypoperfused volume (MTT) of 33 mL without evidence of CCD. The patient received intravenous thrombolysis and was discharged with a NIHSS score of 0. At the 3 month follow-up, the mRS was 0, indicating complete recovery without residual deficits. **B** A 44-year-old woman presented to the emergency department one hour after the onset of left-sided sensorimotor symptoms (baseline NIHSS: 16). Imaging revealed an occlusion of the right M1 segment of MCA, and CT perfusion demonstrated a hypoperfused volume (MTT) of 93 mL with evidence of mild-to-moderate CCD. The patient underwent intravenous thrombolysis followed by mechanical thrombectomy, achieving complete recanalisation (mTICI 3). She was discharged with a final NIHSS score of 0, and at the 3 month follow-up, the mRS was 0, indicating full recovery without residual deficits. **C** A 74 year-old woman presented to the emergency department two and a half hours after the onset of left hemiplegia and dysarthria (baseline NIHSS: 22). Neuroimaging revealed occlusion of the right internal carotid artery (ICA) extending to the M1 segment of the middle cerebral artery (MCA). CT perfusion demonstrated a hypoperfused volume (MTT) of 190 mL with evidence of moderate-to-severe CCD. The patient underwent intravenous thrombolysis followed by mechanical thrombectomy, achieving partial reperfusion (mTICI 2a). She was subsequently transferred to a rehabilitation facility with an NIHSS score of 15. At the 3 month follow-up, the mRS was 5, with persistent motor deficits

The supratentorial hypoperfused volume (MTT) was found to be an independent predictor of CCD, confirming our previous findings [18]. These results suggest that crossed cerebellar diaschisis is more likely to develop in the setting of extensive supratentorial hypoperfusion, where disruption of cortico-ponto-cerebellar pathways becomes more probable. Furthermore, the higher frequency of lobar involvement across increasing CCD severity strengthens the association between lesion extent and the development of diaschisis [30, 31]. Figure 2 shows three emblematic clinical cases based on the absence, mild to moderate presence, and moderate to severe presence of CCD.

Regarding sex distribution, our analysis identified female sex as an independent predictor of presence of CCD. This finding suggests a possible sex-related susceptibility to CCD. Emerging evidence indicates sex-related differences in brain connectivity, particularly within cortico-cerebellar networks, which tend to be more pronounced in males [32]. In men, stronger structural and functional coupling between the cerebral cortex and cerebellum has been observed, whereas in women, relatively reduced cortico-cerebellar connectivity could render these pathways more vulnerable to disruption following cortical injury. However, current evidence remains limited, underscoring the need for further connectome studies specifically designed to explore sex-related variability in cerebral-cerebellar network integrity after acute cerebrovascular events.

In our cohort, higher baseline NIHSS scores were significantly, although weakly, associated with higher grade of CCD, suggesting a relationship between initial stroke severity and the grade of CCD. This observation is consistent with our previous findings [18]. However, the literature on this association remains heterogeneous, with some studies reporting CCD as an independent correlate of stroke severity [33], while others have found no significant correlation [17].

Although CCD was associated with a higher prevalence of unfavorable functional outcomes at 3 months (mRS 3–6) in univariate analysis, this association did not remain significant in multivariable analysis, suggesting that CCD observed at admission may reflect overall stroke severity rather than independently influencing clinical outcome. In this context, this finding may also be explained with the predominant impact of reperfusion therapy, particularly endovascular treatment with successful recanalization, which represents the main determinant of outcome in patients with LVO. The severity of subacute CCD should be further investigated to better evaluate its evolution over time and to clarify its potential predictive value for clinical outcomes. The subgroup analysis in patients without neurological improvement (ΔNIHSS ≤ 5), identified the CCD as an independent predictor of unfavorable outcome, suggesting that its clinical relevance may become more apparent in selected subpopulations with limited recovery.

Future research should aim to characterize the specific supratentorial regions associated with perfusion alterations and their corresponding cerebellar counterparts. Increasing evidence supports a somatotopic organization within the cortico-cerebellar network, in which cortical motor areas are functionally connected to cerebellar motor regions, and higher-order associative cortical areas are linked to the non-motor, associative territories of the posterior cerebellar lobe [34–36]. Understanding these topographical relationships could provide deeper insight into the mechanisms underlying CCD and its clinical implications. Furthermore, functional disconnection patterns following acute stroke may also involve the hemisphere contralateral to the lesion, as shown in previous studies [37, 38].

This study has several limitations that should be acknowledged. First, it is a retrospective analysis, which introduces the potential for unmeasured confounders, and it involves a patient cohort from a single Stroke Unit. The absence of a control group represents an additional limitation, as it precludes a formal evaluation of the specificity of CT perfusion findings related to CCD. The qualitative and semi-quantitative assessment of diaschisis relied on visual interpretation, which may introduce observer bias, and a fully quantitative analysis of CCD was not performed. Another limitation of this study is the performance of multiple statistical comparisons, including subgroup and exploratory analyses, which may increase the risk of type I error. Furthermore, the specific anatomical localization of diaschisis was not systematically analyzed, and no perfusion follow-up imaging was available to evaluate its temporal evolution.

Despite these limitations, this study also presents several strengths. First, this study includes a large and homogeneous cohort of patients with LVO stroke, all assessed in the hyperacute phase using CT perfusion under uniform inclusion criteria, ensuring consistent and timely data acquisition. The evaluation of CCD combined qualitative visual assessment and semi-quantitative volumetric analysis, allowing the detection and grading of even subtle perfusion abnormalities as absent, mild–moderate or moderate–severe.

## Conclusion

In conclusion, perfusion alterations consistent with CCD were detected on hyperacute CTP in a high proportion of patients with LVO stroke (84.4%). Female sex and larger supratentorial hypoperfused volumes were independent predictors of CCD. Although CCD was correlated with functional outcome, it was not an independent predictor. These findings support the hypothesis that the occurrence of CCD in the hyperacute phase may reflect a process of functional disconnection.

## Supplementary Information

Below is the link to the electronic supplementary material.

Supplementary file1 (DOCX 92 KB)

## Data Availability

The data will only be made available from the corresponding author upon reasonable request.

## Abbreviations

CCD: Crossed cerebellar diaschisis
IS: Ischemic stroke
CTP: Computed tomography perfusion
MTT: Mean transit time
