# Supplementary material for: Crossed cerebellar diaschisis on CT perfusion in large vessel occlusion stroke: early predictors and clinical relevance in the hyperacute phase
Source: J Neurol. 2026 Mar 24;273(4):229. doi: 10.1007/s00415-026-13750-z (PMC13013197; doi:10.1007/s00415-026-13750-z)

**Supplemental Table 1.** Comparison of demographics and clinical characteristics of patients with mRS 0-2 vs mRS 3-6 at 3 months. Data are presented as medians (IQR), and frequencies when appropriated.

|  | **mRS 0-2 n=101** | **mRS 3-6 n=155** |
| --- | --- | --- |
| **Demography**  Age (years)  Females  **Comorbidities** [n (%)]  Atrial fibrillation  Diabetes mellitus  Arterial hypertension  Smoke  Dyslipidaemia  Chronic heart failure  Coronaropathy  Peripheral vascular disease  Chronic kidney disease  Cognitive impairment  Previous ischemic stroke    **TOAST classification** [n (%)]  Cardioembolic (CE)  Large artery atherosclerosis (LAA)  Undetermined causes (SUC)  Other determined causes (ODC)  Small vessel occlusion (SVO)  **Stroke severity and clinical outcomes**  NIHSS at baseline  NIHSS at discharge  NIHSS at discharge ≤ 5  Pre-stroke mRS  One-year mortality  Length of hospitalization  **Reperfusion treatment** [n (%)]  Thrombectomy  Thrombolysis  Thrombolysis and thrombectomy  Recanalization score  TICI < 2b  TICI 2b-3  Timing  Time to door (hours)  Time to first CTP (hours)  Time to end of thrombectomy (hours)  **Neuroimaging**  NECT ad admission  ASPECTS [median (IQR)]  CTA at admission  Collateral score [median (IQR)]  CTP at admission           Hypoperfused volume (mL)  Core volume (mL)  NECT at follow-up  Final infarct volume (mL)  Side  Right  Left              Cerebral infarcted area  Affected cerebral lobes  **Diaschisis** [n (%)]  Absent  Present  Severity  Mild to moderate  Moderate to severe | 75 (64-80)  51 (50.5)  44 (43.6)  9 (8.9)  65 (64.4)  25 (24.8)  54 (53.5)  7 (6.9)  10 (9.9)  10 (9.9)  8 (7.9)  5 (5)  6 (5.9)  50 (49.5)  14 (13.9)  26 (25.7)  11(10.9)  0 (0.0)  9 (5-13)  1 (0-2)  96 (95.1)  0 (0-0)  6 (5.9)  9 (5-13)  12 (11.9)  21 (20.8)  62 (61.4)  4 (5.4)  70 (94.6)  1.23 (0.53-2.44)  1.55 (1.19-3.01)  2.29 (2.06-2.53)  9 (9-10)  2 (1-3)  74.3 (50.1-136.9)  8.7 (4-21.9)  4.4 (0-15)  49 (48.5)  52 (51.5)    1 (1-2)  22 (21.8)  79 (78.2)  68 (67.3)  11 (10.9) | 81 (74.5-85)  101 (65.2)  73 (47.1)  38 (24.5)  129 (83.2)  19 (12.3)  80 (51.6)  20 (12.9)  21 (13.6)  23 (14.8)  23 (14.8)  17 (11)  14 (9)  65 (41.9)  26 (16.8)  54 (34.8)  10 (6.4)  0 (0.0)  18 (12.5-22)  13 (5.5-19.5)  97 (62.6)  0 (0-1)  79 (51)  9 (4-18.5)  23 (14.8)  23 (14.8)  85 (54.8)  41 (38)  67 (62)  1.30 (0.59-2.52)  1.58 (1.22-3.12)  2.40 (2.04-3.19)  9 (7.75-10)  1 (1-2)  130.7 (79.5-190.9)  22.1 (9-58.5)  41.5 (8.3-156.3)  61 (39.4)  94 (60.7)  3 (2-5)  18 (11.6)  137 (88.4)  110 (70.9)  27 (17.4) |

**Supplemental Table 2.** Logistic multivariate regression for prediction of poor functional outcome at 3 months (mRS 3-6)

| **Variables** | **OR** | **Cl – 95%** | ***p-values*** |
| --- | --- | --- | --- |
| **Female sex** | 1.029 | 0.919 - 1.151 | 0.616 |
| **Age** | 1.008 | 1.003 - 1.013 | **0.002** |
| **NIHSS at admission** | 1.031 | 1.022 - 1.041 | **<0.001** |
| **Diabetes mellitus (DM)** | 1.178 | 1.031 - 1.347 | **0.016** |
| **Hypertension (HTN)** | 1.067 | 0.940 - 1.211 | 0.308 |
| **Chronic heart failure (CHF)** | 1.123 | 1.009 - 1.250 | **0.032** |
| **Hypoperfused volume (MTT)** | 1.001 | 0.999 - 1.001 | 0.649 |
| **Core volume (CBV)** | 1.001 | 0.999 - 1.001 | 0.429 |
| **Crossed cerebellar diaschisis** | 1.065 | 0.925 - 1.226 | 0.374 |

**Notes:** Multivariate analysis for prediction of poor functional outcome. Bold values for *p* < 0.05.

**Supplemental Table 3.** Logistic multivariate regression for prediction of poor functional outcome at 3 months (mRS 3-6) in patients with CCD

| **Variables** | **OR** | **Cl – 95%** | ***p-values*** |
| --- | --- | --- | --- |
| **Age** | 1.009 | 1.004 - 1.015 | **<0.001** |
| **NIHSS at admission** | 1.027 | 1.018 - 1.037 | **<0.001** |
| **Diabetes mellitus (DM)** | 1.160 | 0.997 - 1.349 | 0.053 |
| **Hypertension (HTN)** | 1.072 | 0.933 - 1.233 | 0.320 |
| **Chronic heart failure (CHF)** | 1.112 | 0.989 - 1.250 | 0.074 |
| **Hypoperfused volume (MTT)** | 1.001 | 0.999 - 1.001 | 0.799 |
| **Core volume (CBV)** | 1.001 | 0.998 - 1.002 | 0.435 |

**Notes:** Multivariate analysis for prediction of poor functional outcome at 3 months (mRS 3-6) in patients with CCD. Bold values for *p* < 0.05.

**Supplemental Table 4.** Logistic multivariate regression for prediction of poor functional outcome (mRS 3-6 at 3 months) in patients without EVT

| **Variable** | **OR** | **Cl – 95%** | ***p-values*** |
| --- | --- | --- | --- |
| **Age** | 1.012 | 1.002 - 1.023 | **0.016** |
| **NIHSS at admission** | 1.032 | 1.017 - 1.047 | **<0.001** |
| **Hypoperfused volume (MTT)** | 1.001 | 0.998 - 1.001 | 0.655 |
| **Core volume (CBV)** | 1.001 | 0.998 - 1.003 | 0.612 |
| **Crossed cerebellar diaschisis** | 1.015 | 0.812 - 1.269 | 0.889 |

**Notes:** Multivariate analysis prediction of poor functional outcome (mRS 3-6 at 3 months) in patients without EVT. Bold values for *p* < 0.05.

**Supplemental Table 5.** Logistic multivariate regression for prediction of poor functional outcome at 3 months (mRS 3-6) in patients without NIHSS improvement during hospitalisation (Δ NIHSS ≤ 5)

| **Variables** | **OR** | **Cl – 95%** | ***p-values*** |
| --- | --- | --- | --- |
| **Female sex** | 1.068 | 0.959 - 1.190 | 0.266 |
| **Age** | 1.003 | 0.998 - 1.009 | 0.161 |
| **NIHSS at admission** | 1.034 | 1.026 - 1.043 | **<0.001** |
| **Diabetes mellitus (DM)** | 1.106 | 0.972 - 1.259 | 0.123 |
| **Hypertension (HTN)** | 1.112 | 0.974 - 1.271 | 0.114 |
| **Hypoperfused volume (MTT)** | 1.001 | 0.999 - 1.001 | 0.464 |
| **Core volume (CBV)** | 0.999 | 0.998 - 1.001 | 0.943 |
| **Crossed cerebellar diaschisis** | 1.164 | 1.005 - 1.350 | **0.042** |

**Notes:** Multivariate analysis for prediction of poor functional outcome at 3 months (mRS 3-6) in patients without NIHSS improvement during hospitalisation (Δ NIHSS ≤ 5). Bold values for *p* < 0.05.

**Supplemental Figure 1.** Violin plot showing the distribution of NIHSS scores at admission in patients without CCD vs. mild-moderate CCD vs. moderate-severe CCD. Abbreviations: CCD: crossed cerebellar diaschisis


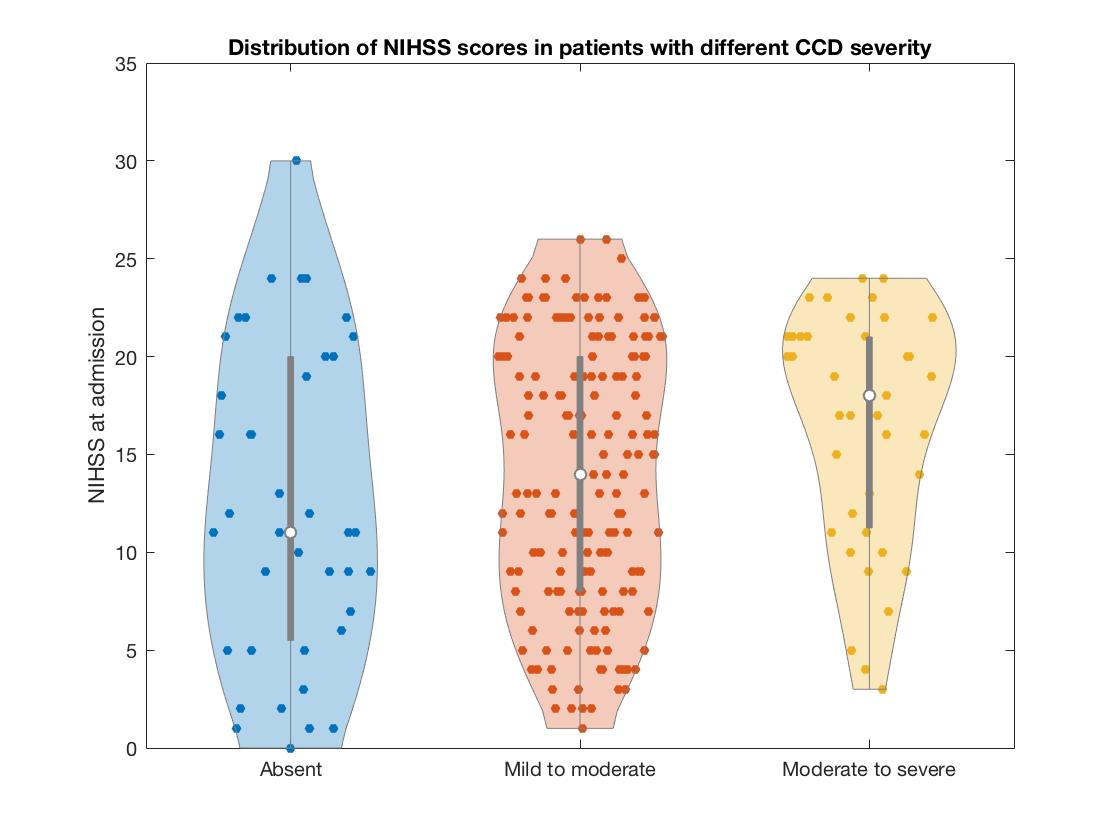

Supplement: Supplementary file 1 — Supplementary file1 (DOCX 92 KB) [file 415_2026_13750_MOESM1_ESM.docx]
